# Dissociation of agency and body ownership following visuomotor temporal recalibration

**DOI:** 10.3389/fnint.2015.00035

**Published:** 2015-05-07

**Authors:** Shu Imaizumi, Tomohisa Asai

**Affiliations:** ^1^Graduate School of Engineering, Chiba UniversityChiba, Japan; ^2^NTT Communication Science Laboratories, NTT CorporationKanagawa, Japan; ^3^Japan Society for the Promotion of ScienceTokyo, Japan

**Keywords:** bodily self-consciousness, action, voluntary movement, proprioceptive drift, sensorimotor processing, time perception, lag adaptation

## Abstract

Bodily self-consciousness consists of one’s sense of agency (I am causing an action) and body ownership (my body belongs to me). Both stem from the temporal congruence between different modalities, although some visuomotor temporal incongruence is acceptable for agency. To examine the association or dissociation between agency and body ownership in the context of different temporal sensitivities, we applied a temporal recalibration paradigm, in which subjective synchrony between asynchronous hand action and its visual feedback can be perceived after exposure to the asynchronous visuomotor stimulation. In the experiment, participants continuously clasped and unclasped their hand while watching an online video of their hand that was presented with delays of 50, 110, 170, 230, 290, and 350 ms. Then, they rated a video of their hand with a delay of 50 ms (test stimulus) with respect to the synchrony between hand action and hand video and the perceived agency over the video. Moreover, proprioceptive drift of participants’ hand location toward the hand video during the exposure was measured as an index of illusory body ownership. Results indicated that perception of agency emerged over the delayed hand video as subjective visuomotor synchrony was recalibrated, but that body ownership did not emerge for the delayed video, even after the recalibration. We suggest that there is a dissociation between agency and body ownership following visuomotor temporal recalibration.

## Introduction

As time is a form of one’s inner sense and self-intuition (Kant, 1781), subjective time has a close connection with our conscious experience, for instance, recognition of the simultaneity between different sensory events and the passage of time. Since time perception arises from acting on and perceiving the external world through our own body, the consciousness of our own actions and body might be shaped by subjective time (Haggard, 2005; Tsakiris et al., 2007). It has been proposed that bodily self-consciousness consists of the following two main components: sense of agency and sense of body ownership (Gallagher, 2000; Tsakiris, 2010). Sense of agency refers to the feeling that I am causing an action and controlling my body (Gallagher, 2000), while sense of body ownership refers to the feeling that my body belongs to me (Tsakiris, 2010). Both components are based on temporal congruence among afferent information that is sourced via different modalities (Haggard, 2005; Tsakiris, 2010).

### Sense of Agency and Body Ownership in Terms of Time

Achieving a sense of agency requires not only voluntary action (Blakemore et al., 1998; Haggard et al., 2002) but also temporal contiguity between the action and outcome (Blakemore et al., 1999; Bays et al., 2005). The internal forward model in the central motor system has been adopted to help explain the origin of agency based on sensorimotor processes (Wolpert et al., 1995; Wolpert, 1997; Frith et al., 2000). This model is based on an efference copy of motor commands from a self-produced action (von Holst and Mittelstaedt, 1950) and predicts sensory feedback of the motor commands before actual afferent feedback. If this prediction mismatches the feedback because of temporal biases, perceived agency will decrease. Thus, delayed visual feedback following voluntary action is hard to attribute to the self (Franck et al., 2001; Asai and Tanno, 2007b; Farrer et al., 2008). Further, an outcome that can be perceived to precede the action is also difficult to attribute to the self (Rohde et al., 2014; Timm et al., 2014), since action commonly precedes sensory outcome (Wegner and Wheatley, 1999). However, a certain amount of temporal discrepancy between voluntary action and its sensory feedback can be acceptable for perceiving a sense of agency (Bays et al., 2005; Miyazaki and Hiraki, 2006; Asai and Tanno, 2007b).

Studies utilizing the rubber hand illusion (RHI) suggest that sense of body ownership is based on temporal contiguity between visuotactile afferent inputs (Botvinick and Cohen, 1998; Tsakiris and Haggard, 2005; Tsakiris et al., 2006) and is spatially plastic, that is, observers can perceive body ownership even toward an external object that is separate from their own body. In the RHI, observers watch a rubber hand being stroked, while their own unseen hand is being synchronously stroked for a short time, and start to feel as if the rubber hand belongs to their own body. Consequently, the RHI will not occur when these strokes are applied asynchronously. In addition, the misattribution of body ownership to the rubber hand can be accompanied by a shift of the proprioceptively felt location of the observers’ hand toward the rubber hand. This shift, known as proprioceptive drift (e.g., Botvinick and Cohen, 1998), can serve as an implicit measure of body ownership and has also been observed in an active version of the RHI when induced by the observers’ physical hand action and its visual feedback on a video screen (Tsakiris et al., 2006). Although the interpretation of this active RHI is still controversial because of the co-occurrence of agency and body ownership under proprioceptive drift (Sanchez-Vives et al., 2010; Kalckert and Ehrsson, 2012), body ownership would inhabit temporally matching sensations among different modalities, including the motor domain (Tsakiris, 2010; Asai, 2015a).

### Temporal Plasticity in Agency and Body Ownership

While previous studies have suggested that both agency and body ownership are innately acquired through congruent temporal matching between modalities, human time perception is known to be plastic. An example of plasticity of time perception is the temporal recalibration, whereby observers exposed to a time lag between bimodal stimuli show a shift in their subjective simultaneity perception between these asynchronous stimuli (Fujisaki et al., 2004; Vroomen et al., 2004). This plasticity should play a key role in compensation for cross-modal latencies, which result from the different neural processing times required by different modalities (Spence and Squire, 2003). The temporal recalibration is also found in visuomotor (Stetson et al., 2006) and audiomotor domains (Heron et al., 2009). These couplings between modalities are especially related to sense of agency, as mentioned above.

Does temporal recalibration, in turn, lead to plasticity in agency and body ownership? Body ownership might not be temporally calibrated or learned, since it is widely accepted that asynchronous visuotactile stimulation for even a few minutes does not elicit the RHI (Botvinick and Cohen, 1998; Tsakiris and Haggard, 2005; Tsakiris et al., 2006; Kalckert and Ehrsson, 2012); however, this is controversial because of a proposal that body ownership can be affected by temporally incongruent visuotactile stimulation (Shimada et al., 2009, 2014). On the other hand, some studies have indicated that agency is temporally plastic, that is, sense of agency can be experienced when learned prediction of the sensory outcome of an action matches the actual outcome (Frith et al., 2000; Blakemore et al., 2002; Bays et al., 2005). Repeatedly experiencing a sensory outcome following an action causes the learning of action–outcome associations in terms of contextual and temporal congruence. As an example of a contextual association, observers can learn to anticipate a tone with a certain pitch that is triggered by a self-initiated keypress (Elsner and Hommel, 2001) and consequently perceive a sense of agency over the tone (Sato and Yasuda, 2005). As an example of a temporal association, Asai and Tanno (2007a), it was suggested that a delay between an action and its feedback can be learned and become acceptable for generating the sense of agency, while there were no learning effects in relation to detection of the delay. Recent studies have suggested that it is hard to perceive a sense of agency over the tone in the context of audiomotor temporal recalibration in which self-initiated sounds are subjectively perceived as preceding keypresses (Timm et al., 2014). Moreover, Keetels and Vroomen (2012) demonstrated a visuomotor temporal recalibration between finger tap and the video of the hand, although this action in a natural situation does not entail delayed visual feedback. However, these authors did not directly examine sense of agency.

### Association and Dissociation between Agency and Body Ownership

Although it can be assumed that visuomotor temporal recalibration modulates sense of agency but not body ownership, how temporal recalibration between a voluntary action and its visual feedback retains a sense of agency and body ownership remains unclear.

While both agency and body ownership are necessary for self-recognition (van den Bos and Jeannerod, 2002), it has been suggested that the two are both associated and dissociated. The active RHI suggests an association, in which voluntary action accompanying a sense of agency integrates distinct body parts into a coherent sense of body ownership (Tsakiris et al., 2006). Other studies applied the active RHI paradigm to compare its effects under conditions with and without agency (e.g., active vs. passive movements) and to investigate the interaction between sense of agency and body ownership (Kalckert and Ehrsson, 2012, 2014a,b; Braun et al., 2014; Asai, 2015a). Recent studies have suggested that sense of agency can override body ownership (Tsakiris et al., 2007), that is, agency over a moving hand image itself generates body ownership toward the image even when there is asynchrony between them (Asai, 2015a).

In contrast, sense of agency and body ownership can be dissociated not only conceptually (Gallagher, 2000; Tsakiris, 2010) and neurally (Tsakiris et al., 2010) but also in terms of spatiotemporal factors. Since the spatial component of visual feedback plays a more crucial role than the temporal component does in eliciting a sense of agency (Farrer et al., 2008), a certain amount of delay of visual feedback could be acceptable for eliciting agency (Bays et al., 2005; Miyazaki and Hiraki, 2006; Asai and Tanno, 2007b). Body ownership, however, requires temporal congruence between inter-sensory stimuli (Botvinick and Cohen, 1998; Tsakiris and Haggard, 2005).

### Present Study

To date, no studies have examined how the temporal component of an action and its feedback modulates sense of agency and body ownership, respectively and simultaneously. This might be because voluntary action always entails both agency and body ownership (Tsakiris, 2010). To address this gap in the literature, we attempted to dissociate agency and body ownership by taking advantage of their different sensitivity to temporal factors, that is, agency allows a relatively broad time window of asynchrony (e.g., Bays et al., 2005) while body ownership does not (e.g., Botvinick and Cohen, 1998). To achieve this, we applied a temporal recalibration paradigm, involving observers recalibrating an asynchrony between their hand action and its visual feedback (Keetels and Vroomen, 2012).

Our two hypotheses regarding dissociation of agency and body ownership were as follows: First, observers will perceive sense of agency over delayed visual feedback of their hand action after visuomotor temporal recalibration. Second, observers will not perceive illusory body ownership toward the delayed visual feedback even after the visuomotor temporal recalibration, because the recalibration does not entail body ownership unless it is elicited by agency.

To test these hypotheses, we conducted an experiment in which participants were exposed to an online video of their hand action with six levels of delay (adaptation stimulus). Then, they rated a hand video with the shortest delay (test stimulus) with respect to the synchrony between hand action and video and the perceived agency over the video. Moreover, proprioceptive drift toward the video image was used as a measure of shift of body ownership during the adaptation phase.

We expected that a greater delay of the adaptation stimulus would result in a stronger temporal recalibration and, consequently, lower ratings of synchrony and agency over the test stimulus, which would be perceived as illusorily preceding the actual hand action. On the other hand, proprioceptive drift was expected to emerge in relation to the adaptation stimulus with minimal delay, but not in relation to delayed adaptation stimuli, because it can be assumed that temporal recalibration does not affect body ownership. Conversely, if temporal recalibration affects body ownership because agency overrides body ownership, substantial proprioceptive drift should be found under all conditions, regardless of the magnitude of adaptation-stimulus delay.

## Materials and Methods

### Participants

Nineteen adults (15 females; mean age 33.21 ± 6.21 years), naïve with respect to the study purpose, participated in return for monetary compensation. All were self-declared right-handed and had normal or corrected-to-normal visual acuity and no neurological or psychiatric illness. Written informed consent was obtained from each participant. This study was approved by the ethical committee of the NTT Communication Science Laboratories and was conducted in accordance with the principles of the Declaration of Helsinki.

### Experimental Setup

**Figure 1:**
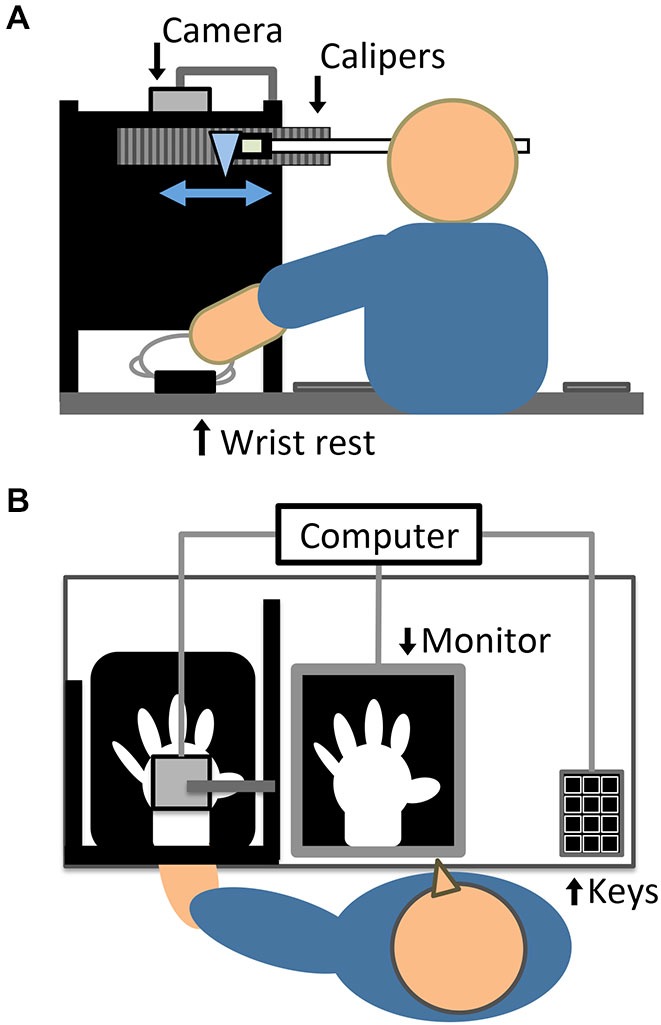
**Experimental setup, depicted in (A) elevated and (B) horizontal views**. In the actual experiment, a black cloth covered the tabletop and participants wore a black cape covering the left arm and shoulder.

The setup (see Figures 1A,B), in which visual feedback of participants’ hand action was provided with and without delay, followed that of previous study which examined active RHI (Asai, 2015a). Participants sat at a table in a semi-dark and quiet room. Their left hand was put on a wrist rest at the edge of the table to prevent the wrist from moving from side to side during the hand action. The distance between the center of the left wrist and the center of participants’ body was approximately 40 cm. The left hand was covered with a white glove to remove morphological cues for self-identification. The dorsum of the left hand was recorded by a color video camera (STC-TC33USB-AS, Sensor Technologies America, Inc.) at 60 frames per second, from 37.5 cm directly above the hand. The tabletop was covered with a black cloth so that the camera captured the hand against a black background. An LED light (LE-H631B, Twinbird Corp.) illuminated the space near the left hand. We targeted the left hand, following previous typical RHI studies (e.g., Botvinick and Cohen, 1998), because it has been suggested that there is a right-hemispheric dominance for body ownership and a stronger RHI effect for the left hand (Ocklenburg et al., 2011).

A program written with Hot Soup Processor version 3.31 (ONION Software) generated video images of the left hand with systematic delays (50, 110, 170, 230, 290, or 350 ms; all including intrinsic transmission delay). The minimum 50-ms delay can be accepted as synchronous feedback because this is below the 150 ms threshold of detecting visuomotor delays (Tsakiris et al., 2006, 2010; Longo and Haggard, 2009). The filmed images were processed and simultaneously displayed on a 23-inch LED monitor (i2353Ph, AOC) with a resolution of 1920 × 1080 pixels and a refresh rate of 60 Hz. A computer (CF-SX1, Panasonic Corp.) with the above program running on Windows 7 was used to control image processing, stimulus presentation, and response collection. The monitor was laid on the table in front of the participants, at approximately 45 cm from the head. The hand image appeared to be almost life-sized on the monitor and was displaced approximately 18 cm rightward from the center of the left wrist. This displacement should be acceptable for inducing proprioceptive drift since the spatial limit in this regard has been reported as 30 cm (Lloyd, 2007). To obstruct the direct view of the left hand and arm, participants placed the left hand behind a black standing screen aligned with the mid-sagittal plane on the table, and wore a black cape covering the left arm and shoulder. A loudspeaker built into the monitor presented an auditory metronome with alternate high and low pure tones (100 beats per minute), in order to have all participants enact and view the same number of hand movements.

During proprioceptive drift measurement, as described below, participants indicated the felt horizontal location of their left wrist by moving from side to side with the right hand a transparent rod positioned before their eyes, which was attached to the arrowhead of digital Vernier calipers (CD-30C, Mitutoyo Corp.). The calipers were attached to a standing screen to the front and left of the participants, at a height of 36 cm. The distance between the point of the arrowhead and the top of participants’ left wrist was approximately 27 cm. The participants could not see any cues for the hand location, such as numbers and marks; however, the experimenter could see numbers indicating the location of the arrowhead in units of 0.01 mm. Larger numbers indicated a location closer to the center of the body. This apparatus (Asai, 2015b) allows for more minute measurement of proprioceptive drift compared to asking the felt hand location by pointing using the opposite hand (Botvinick and Cohen, 1998) or verbally reporting a number on a ruler (Tsakiris et al., 2006), which have been traditionally used.

### Procedures

**Figure 2:**
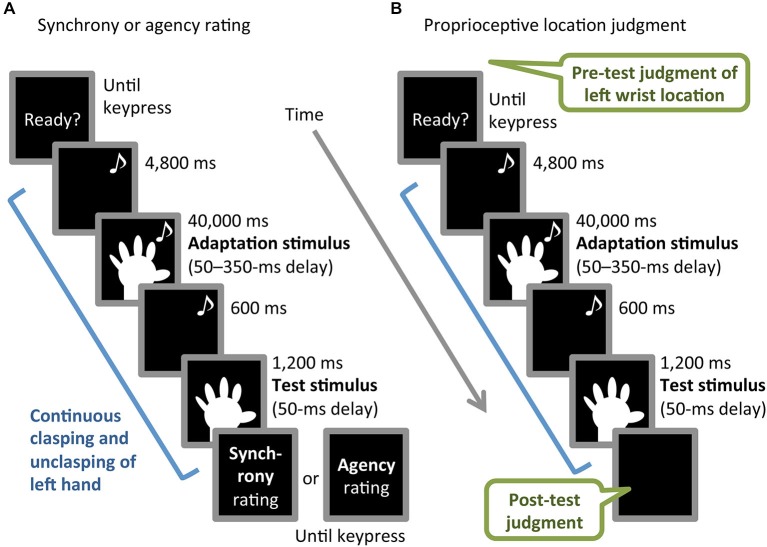
**Schematic illustration of (A) synchrony or agency rating task and (B) proprioceptive location-judgment task**. Musical notes indicate periods with an auditory metronome.

The experiment consisted of three blocks, in which participants performed synchrony rating, agency rating, or proprioceptive location-judgment tasks (Figures 2A,B). Each of the three tasks was based on a common procedure comprising adaptation and test phases and involving continuous left-hand action, as described below. The order of the blocks was counterbalanced across participants. The inter-block interval was approximately an hour and a half. Participants performed an unrelated filler task during the interval in order to obstruct potential carry-over effects.

The common procedure started with the metronome to cue the continuous clasping and unclasping of participants’ left hand at 0.83 Hz, triggered by participants’ pressing the enter key with their right hand. The participants viewed the monitor throughout the task. For the first 4,800 ms, the monitor presented a black screen while the participant clasped and unclasped the left hand. For the next 40,000 ms, the video image of the left hand with one of the six delays (50–350 ms) was presented (adaptation stimulus). Finally, the left-hand image with a 50-ms delay was presented for 1,200 ms without the metronome (test stimulus), preceded by a 600-ms black screen. During the test stimulus period, the participants were instructed to keep the same pace without the metronome, in order to avoid introducing the cue of the temporal proximity between test stimulus and left-hand movement, which might counteract the recalibration effect. When the test stimulus period ended, participants stopped the hand movement and the monitor presented questions and/or instructions regarding each task. The inter-trial interval was 5,000 ms in all blocks.

In the synchrony rating task, the participants performed the common procedure once, then answered the following question by pressing number keys (1–9), corresponding to a 9-point Likert scale ranging from 1 (not at all) to 9 (extremely): “To what extent did you feel that the motion of the hand image presented just before was incongruent with your hand movement?” The question and scale were presented on the monitor until the keypress was made. The synchrony rating task block consisted of 2 practice and 18 main trials (three repetitions of each of six adaptation-stimulus delay conditions), in which the order of the delay conditions was randomized.

The agency rating task was identical to the synchrony rating task, except for the following difference. The monitor presented the question “To what extent did you feel that the hand image presented just before was your hand?” At the beginning of this block, we gave participants the instruction regarding the question: “Answer the question in view of whether the movement of the hand image can be felt as self- or other-controlled, although the image was of the participant’s hand.”

The proprioceptive location-judgment task consisted of pre-test proprioceptive location judgment, the common procedure, and post-test location judgment. In the pre-test judgment, participants had their eyes open and were asked to indicate the proprioceptively felt location of the center of their unseen left wrist by looking at and moving the arrowhead of the calipers with no time constraint. At this time, the hand image on the monitor was outside the participants’ visual field. The experimenter recorded the location indicated on a digital display of the calipers. This location judgment was repeated twice for precise measurement, before the common procedure was completed once. The participants were instructed not to intentionally shift the left wrist location during adaptation-stimulus and test-stimulus periods. Immediately after the test-stimulus presentation, at the end of the common procedure, post-test location judgment was performed in the same manner as that of the pre-test. The block with the location judgment-task consisted of 2 practice and 12 main trials (two repetitions of each six adaptation-stimulus delay), in which the order of the delay conditions was randomized. Proprioceptive drift was calculated by subtracting the mean of two pre-test judgments from that of the post-test judgments. A positive value of the drift indicated that illusory body ownership transferred to the hand image.

### Data Analysis

For consistency of synchrony and agency ratings, the synchrony rating score was reversed so that a score of 1 (not at all feeling that the motion of test stimulus was incongruent with the hand movement) was transformed to 9. Synchrony and agency ratings and proprioceptive drift in each delay condition were averaged separately for each participant. Statistical analyses were performed using SPSS version 22.0 (IBM Corp.), with the significance level set at *p* < 0.05.

A simple regression analysis with adaptation-stimulus delay as an independent variable and synchrony rating, agency rating, and proprioceptive drift as dependent variables was conducted to examine whether adaptation-stimulus delay predicts the synchrony and agency ratings for test stimulus and proprioceptive drift toward the hand image. We reported non-adjusted coefficient of determination (*R*^2^) and standardized partial regression coefficient (β) and tested the significance of the slope of regression by *t* test. If temporal recalibration emerges and leads to agency, as proposed in the first hypothesis, there will be negative regression slopes for the synchrony and agency ratings, indicating that larger adaptation-stimulus delay results in a stronger temporal recalibration and, consequently, lower synchrony and agency ratings for the stimulus with minimal delay. On the other hand, if temporal recalibration does not lead to body ownership, as proposed in the second hypothesis, there will also be a negative regression slope for proprioceptive drift, indicating that proprioceptive drift does not emerge in relation to delayed adaptation stimuli.

For the case that the second hypothesis was supported, we examined the conditions in which proprioceptive drift occurred by one-tailed *t* tests to confirm whether the proprioceptive drift in each delay condition was higher than zero. The effect size was reported as* r* (Cohen, 1988).

For further testing, we performed an ANOVA for synchrony rating, agency rating, and proprioceptive drift to reveal the effect of delay (six adaptation-stimulus delays) and the differences between conditions. For these ANOVAs, the Huynh-Feldt correction was applied to the degrees of freedom when the sphericity assumption was violated. The effect size was reported as *η*^2^ (Cohen, 1988). Dunnett’s multiple comparisons were performed on synchrony rating, agency rating, and proprioceptive drift to compare the minimum 50-ms delay condition with the other five conditions in order to examine how much delay is crucial for visuomotor temporal recalibration.

Finally, we calculated Pearson’s product-moment correlation coefficients (*r*) for the relationships between synchrony rating, agency rating, and proprioceptive drift. These correlations examined the concurrence of temporal recalibration, sense of agency, and body ownership.

## Results

Six participants were excluded from the analysis. Five failed to experience any visuomotor temporal recalibration, because each of their mean synchrony ratings across conditions were 1 standard deviation (SD) above the mean across participants (mean = 7.62, SD = 1.24), indicating a rating of 9 (strong feeling of congruence between test stimulus and hand action) for almost all trials. One participant showed a highly positive slope of the linear regression of agency ratings that was 1 SD above the mean across participants (mean = −0.19, SD = 0.42), indicating increased agency ratings for longer delays after the recalibration possibly due to misunderstanding the task.

**Figure 3:**
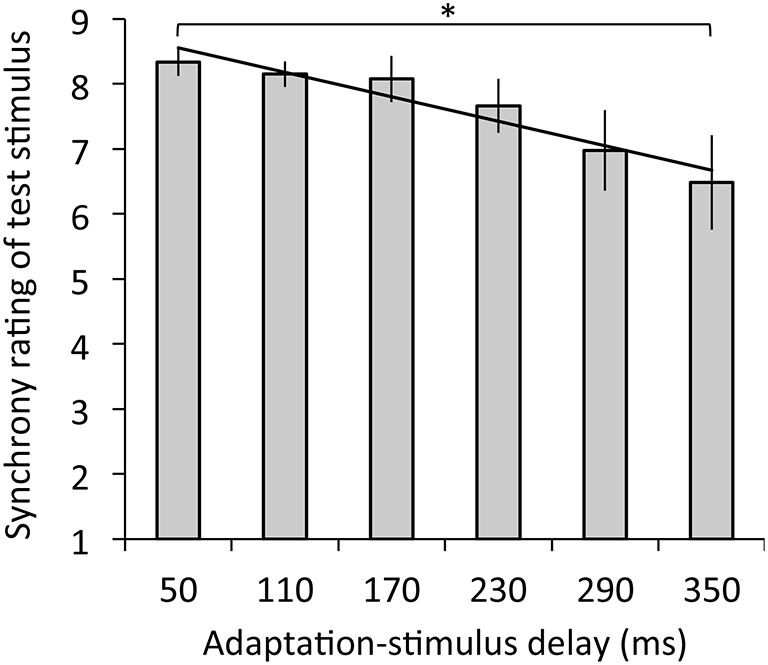
**Synchrony ratings for test stimulus with the 50-ms delay in relation to mean ratings as a function of adaptation-stimulus delay with a significant regression line**. A higher rating means a stronger subjective synchrony between participants’ hand action and the hand image. Error bars denote ±1 standard error of the mean. Asterisk indicates a significant difference between conditions (*p < 0.05).

Figure 3 depicts the results of synchrony rating. The regression analysis revealed that adaptation-stimulus delay significantly explained synchrony rating, *R*^2^ = 0.14, *β* = −0.39, *t*_(77)_ = 3.45, *p* < 0.01. The ANOVA revealed a significant main effect of delay on synchrony rating, *F*_(1.66,19.94)_ = 4.56, *p* < 0.05, *η*^2^ = 0.28. Multiple comparisons revealed that synchrony ratings were significantly decreased under the 350-ms delay condition relative to the 50-ms delay (*p* < 0.05).

**Figure 4:**
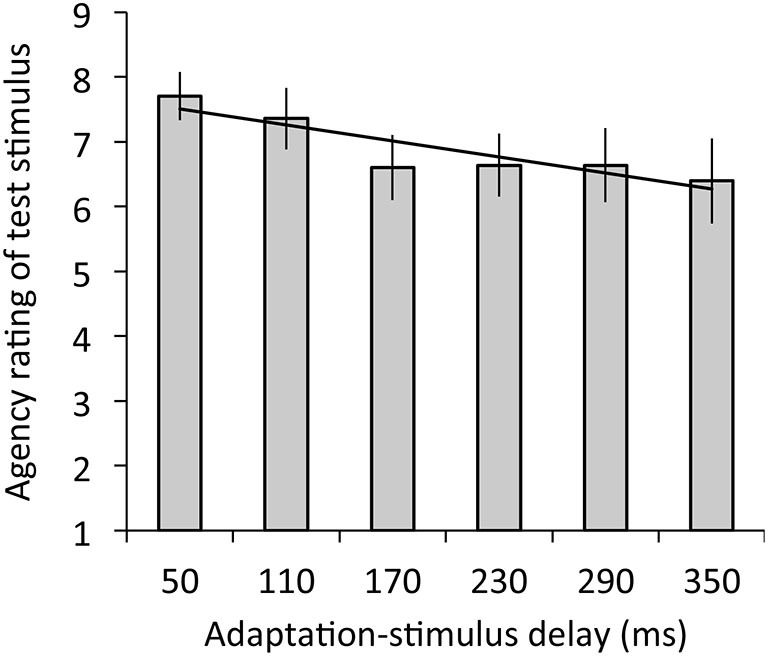
**Agency ratings for test stimulus with the 50-ms delay in relation to mean ratings as a function of adaptation-stimulus delay with a significant regression line**. A higher rating means a stronger sense of agency over the hand image. Error bars denote ±1 standard error of the mean.

Figure 4 depicts the results of agency rating. The regression analysis revealed that adaptation-stimulus delay significantly explained agency rating, *R*^2^ = 0.05, *β* = −0.28, *t*_(77)_ = 2.03, *p* < 0.05. The ANOVA revealed a significant main effect of delay on agency rating, *F*_(2.98,35.72)_ = 3.39, *p* < 0.05, *η*^2^ = 0.22.

**Figure 5:**
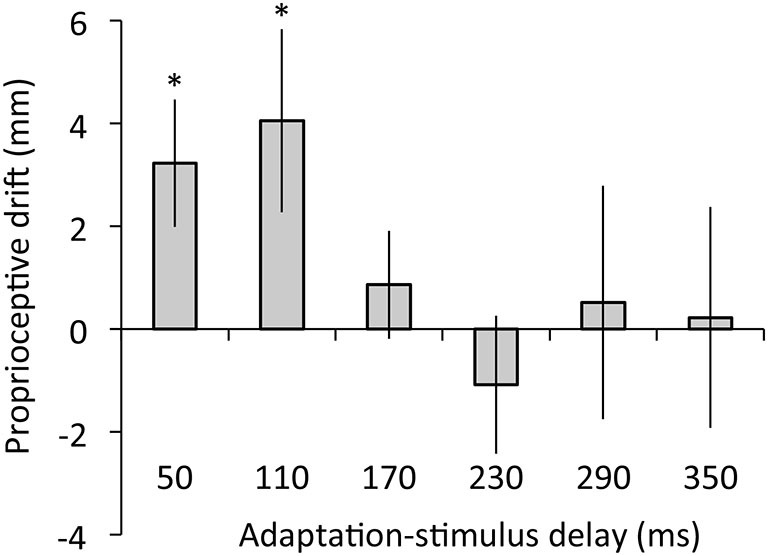
**Proprioceptive drift as a function of adaptation-stimulus delay**. Error bars denote ±1 standard error of the mean. Asterisks indicate values that are significantly different from zero (*p < 0.05).

Figure 5 depicts the results of proprioceptive drift. The regression analysis revealed that adaptation-stimulus delay did not explain proprioceptive drift, *R*^2^ = 0.05, *β* = −0.22, *t*_(77)_ = 1.95, *p* = 0.06. The ANOVA revealed no main effect of delay on proprioceptive drift, *F*_(5,60)_ = 1.30, *p* = 0.28, *η*^2^ = 0.10. However, *post hoc* *t* tests confirmed that proprioceptive drift was significantly higher than zero under 50- and 110-ms delay conditions (50 ms: *t*_(12)_ = 2.60, *p* < 0.05, *r* = 0.60; 110 ms: *t*_(12)_ = 2.28, *p* < 0.05, *r* = 0.55) but not under the other conditions, *t*s < 0.83, *p*s > 0.43, *r*s < 0.24.

As for the correlations between synchrony rating, agency rating, and proprioceptive drift, we found a significantly positive correlation between synchrony and agency ratings (Pearson’s *r*_(78)_ = 0.44, *p* < 0.01), whereas there were no correlations with proprioceptive drift (synchrony and drift: *r*_(78)_ = 0.15, *p* = 0.20; agency and drift: *r*_(78)_ = 0.11, *p* = 0.33).

## Discussion

### Summary of Results

The present study examined sense of agency and body ownership in relation to one’s own hand image following temporal recalibration between physical hand action and the hand image, with temporal discrepancy varying between 50 and 350 ms. Our results indicated two main findings.

First, participants had difficulty in perceiving synchrony for the synchronous visual feedback of hand action following exposure to the delayed visual feedback. Our results revealed that the magnitude of temporal discrepancy between hand action and hand image (adaptation stimulus) predicted perceived synchrony between hand action and hand image with the minimal delay (test stimulus) after the adaptation phase, although this specific difference was found only between synchrony ratings under the 50- and 350-ms delay conditions. These findings suggest the occurrence of a visuomotor temporal recalibration similar to a recent study reporting temporal recalibration between finger taps and their video image (Keetels and Vroomen, 2012). Furthermore, our regression analysis indicated that adaptation-stimulus delay also predicted agency over the hand image with the minimal delay, suggesting that sense of agency over the synchronous hand image decreased with temporal recalibration in a similar manner to the perceived synchrony. Recently, a shift of the criterion for judging agency has been reported to occur in the same manner as that for temporal order judgment following audiomotor temporal recalibration (Timm et al., 2014). In principle, following temporal recalibration, observers illusorily perceive the outcome as that preceding the action (Stetson et al., 2006). Therefore, we speculate that because our participants were likely to perceive the preceding video image of their own hand after temporal recalibration, they consequently had difficulty in attributing the hand image to the self (Rohde et al., 2014). Taken together, our results extend previous findings by suggesting that the visuomotor temporal recalibration in voluntary action entails a recalibration of sense of agency over one’s own body.

Second, our results indicated that proprioceptive drift was not predicted by adaptation-stimulus delay and occurred only under 50- and 110-ms delay conditions, suggesting that proprioceptive drift emerged only in the context of synchronously moving visual feedback, regardless of visuomotor temporal recalibration. In addition, the results showed no correlation of proprioceptive drift with either synchrony or agency rating. These results suggest that visuomotor temporal recalibration does not entail recalibration of body ownership toward the external object (i.e., video image of the hand), as we hypothesized. In line with previous RHI studies (Botvinick and Cohen, 1998; Tsakiris and Haggard, 2005), our participants did not show proprioceptive drift toward an asynchronously moving image of their hand action (i.e., adaptation stimulus with 170- to 350-ms delay). However, the proprioceptive drift was found under 50- and 110-ms delay conditions probably because these delay values of the video image were below 150 ms and acceptable for attributing the video image to the self (Blakemore et al., 1999; Franck et al., 2001; Longo and Haggard, 2009).

### Dissociation between Agency and Body Ownership

Our two main findings suggest a dissociation between sense of agency and body ownership following temporal recalibration. This seems reasonable in the light of previous studies that separately examined agency and body ownership and showed that agency varies with temporal recalibration (Timm et al., 2014), while body ownership is disrupted on a continuous basis by asynchronous stimulation (e.g., Botvinick and Cohen, 1998). In contrast, other studies have suggested that, in a situation without temporal recalibration, agency itself can elicit body ownership (e.g., Asai, 2015a). However, given that in the present study, proprioceptive drift did not emerge under conditions with a 170-ms or larger adaptation-stimulus delay, while there was perception of agency under these conditions, it can be assumed that agency cannot override body ownership following temporal recalibration. We speculate that this dissociation may stem from distinct neural networks underlying the independence model (Tsakiris et al., 2010), which posits that agency and body ownership are qualitatively different. On the other hand, it can also be said that the dissociation comes from differences in the extent to which agency and body ownership depend on temporal congruence for their emergence. Some visuomotor temporal incongruence is acceptable for sense of agency (approximately 380 ms: Rohde et al., 2014; 500 ms: Asai and Tanno, 2007b; 1,000 ms: Miyazaki and Hiraki, 2006), while body ownership allows shorter inter-sensory temporal incongruence (e.g., Botvinick and Cohen, 1998; Tsakiris and Haggard, 2005; Tsakiris et al., 2006; for exceptions, see also Shimada et al., 2009, 2014), perhaps because agency can emerge during a conceptual evaluation stage even when substantial sensorimotor temporal incongruence prevents the self-attribution of sensory feedback for action (Synofzik et al., 2008). Thus, agency can flexibly emerge based on the subjective time window, even if subjective visuomotor synchrony has been illusorily recalibrated. Conversely, body ownership may reject physical temporal incongruence based on sensorimotor processing regardless of temporal recalibration. Further investigation will be needed to understand the origin of this dissociation.

### Individual Differences in Temporal Recalibration

Why did five of our participants not show evidence of temporal recalibration? Although a previous study has demonstrated individual differences in the temporal distance between audiovisual stimuli required for the subjective synchrony under a situation without temporal recalibration (Stone et al., 2001), there has been, to our knowledge, no evidence of individual differences in the adaptation to asynchrony between bimodal stimuli (i.e., temporal recalibration). One possible explanation for the results of these five participants may come from individual differences in monitoring of the action (Fourneret and Jeannerod, 1998) revealed by using a traditional line-drawing task (Nielsen, 1963), in which observers trace sagittal lines on a tablet with their hand using a stylus. The hand was hidden by a mirror, in which they viewed the lines, which either corresponded to or were biased away from the actual trajectory. Fourneret and Jeannerod (1998) showed that some participants misperceived the direction of their hand movement in the direction opposite to the bias, while the others perceived the correct direction. They discussed these group differences with respect to the following two ways of action monitoring: First, reliance on implicit central monitoring of mismatches results from a comparison between the predicted sensory consequence based on the motor commands and the actual consequence (Held, 1961; Wolpert et al., 1995). Second, coinciding the felt position of the arm with the biased visual feedback cancels out the conflict (Harris, 1963). Fourneret and Jeannerod’s participants, who were not perturbed by the biased visual feedback, may have used the former way to a greater extent, while the others may have relied more on the latter way.

Our participants, who did not show visuomotor temporal recalibration, might have applied the former way, by strictly monitoring the action and its sensory feedback. In our experiment, proprioceptive feedback always matched the action, while visual feedback was almost always mismatched (i.e., was delayed). Thus, they may have ignored visual feedback that temporally mismatched the action in order not to attain temporal recalibration. However, it still remains unclear whether individuals use different ways to monitor the action involving temporally mismatching feedback, and whether there are individual differences in the temporal recalibration effect. Future studies should examine these unresolved issues and find the origin of individual differences, such as interoceptive sensitivity, which predicts malleability of body ownership stemming from multisensory stimulation (Tsakiris et al., 2011) and personality, for instance, schizotypy, which can cause an altered prediction of one’s own action (Asai and Tanno, 2007b; Asai et al., 2008).

### Limitations

Our study has three limitations. First, we cannot say to what degree visuomotor temporal recalibration occurred, since we did not apply the method of constant stimuli to measure the shift of point of subjective simultaneity, which has been traditionally used in temporal recalibration studies (Fujisaki et al., 2004; Vroomen et al., 2004; Stetson et al., 2006; Heron et al., 2009; Keetels and Vroomen, 2012). Moreover, we could not examine just noticeable differences indicating the temporal window of the subjective simultaneity between multimodal stimuli affected by temporal recalibration (Navarra et al., 2005; Winter et al., 2008), especially when involving naturalistic feedback such as hand video (Keetels and Vroomen, 2012). Although future study should overcome this limitation, our methods allowed us to examine the relationship between agency and body ownership under virtually the same temporal-recalibrated situation, by using the common procedure with a long-term adaptation phase to induce recalibration and illusory body ownership.

Second, voluntary hand action itself may have affected temporal recalibration. Observers can perceive a shortened duration of the interval between voluntary action and its outcome, so-called “intentional binding”, which can serve as an implicit measure of sense of agency (Haggard et al., 2002; Engbert et al., 2007; Moore and Obhi, 2012). In the present study, participants’ voluntary hand action may have entailed intentional binding to some extent. Furthermore, natural visual feedback of one’s hand action (i.e., the hand video) may have provided a potential cue for perceiving agency and increased intentional binding. Consequently, the perceived discrepancy between the hand action and the video may have been lessened. If so, it can be assumed that there was difficulty in ensuring that visuomotor temporal recalibration emerged. Keetels and Vroomen (2012) also discussed this, with their results showing a large proportion of “synchronous” responses to physically delayed visual feedback of finger taps in terms of intentional binding.

Finally, care should be taken to interpret our results by measuring only explicit agency (i.e., rating) and implicit body ownership (i.e., proprioceptive drift), since explicit and implicit measures do not necessarily coincide (Shimada et al., 2009; Rohde et al., 2011; Braun et al., 2014). For instance, under certain conditions, feeling of agency and intentional binding (implicit agency) cannot necessarily correlate (Braun et al., 2014), and a body ownership questionnaire can detect the emergence of illusory body-ownership, even when proprioceptive drift does not occur (Shimada et al., 2009). On the other hand, using both explicit and implicit measures of agency and body ownership, previous studies have suggested that other types of dissociation are caused by *spatial* factors and motor commands (Kalckert and Ehrsson, 2012; Braun et al., 2014). It would be beneficial if both agency and body ownership were measured explicitly and implicitly. However, we did not find any implicit measurement of agency (e.g., intentional binding paradigm: Haggard et al., 2002) suitable for the current experiment inducing temporal recalibration. Thus, our findings are limited to the specific case where explicit agency and implicit body ownership are measured. Future research should explore the explicit and implicit aspects of the dissociation between agency and body ownership following temporal recalibration by resolving the methodological issues mentioned above.

### Conclusion and Outlook

In conclusion, our results indicated that temporal recalibration between voluntary action and the asynchronous hand-video image entailed a sense of agency over the hand image but did not elicit body ownership toward the hand image. We suggest that the dissociation between agency and body ownership might be due to different sensitivities for generating agency and body ownership. Future studies should examine the interaction between time perception and bodily self-consciousness in other modalities, such as in the auditory domain (i.e., vocalization) and in clinical populations, especially in people with phantom limb pain following amputation (Ramachandran and Hirstein, 1998). Both sense of agency (Cole et al., 2009) and ownership (Lenggenhager et al., 2014) over a prosthesis and virtual limb can play a key role in alleviating pain. Given that the sensorimotor mechanism for generating agency over an online hand image remains for the phantom limb (Imaizumi et al., 2014), further investigation would allow us to uncover the mechanism of embodiment to the external proxies.

## Author Contributions

SI, TA designed the experiment. SI performed the experiment. SI, TA analyzed the results and wrote the manuscript. All authors approved the final version of the manuscript.

## Conflict of Interest Statement

The authors declare that the research was conducted in the absence of any commercial or financial relationships that could be construed as a potential conflict of interest.

## Abbreviations

ANOVA: analysis of variance
RHI: rubber hand illusion
SD: standard deviation.
